# S3-1 Explorations of physical activity programmes among people with chronic diseases

**DOI:** 10.1093/eurpub/ckad133.014

**Published:** 2023-09-11

**Authors:** Chair(s):, Alexandre Mouton, Suzanne McDonough, Anne Vuillemin

**Affiliations:** Sport Sciences Department, University of Liege, Belgium; Population Health and Health Services Research Theme, School of Physiotherapy, RCSI University of Medicine and Health Sciences, Dublin, Ireland; Université Côte d’Azur, LAMHESS, France

## Abstract

Regular physical activity throughout life contributes to optimized mental, social, and physical health of individuals. Yet, a significant ratio of the global population does not meet WHO’s recommended levels of physical activity (WHO, 2022). Promoting physical activity is particularly important among people with chronic diseases, as those longterm noncommunicable conditions are the world’s leading cause of mortality and represent an increasing burden on healthcare systems, economic development, and wellbeing of a large proportion of the population, particularly those aged 50 or above.

When a chronic disease is first identified, the health care sector is generally well organized to provide healthcare management of the condition itself, but there isn’t a consistent approach to adopt and maintain a healthy lifestyle as people move back into community settings. Promoting physical activity as part of routine patient care is a particularly important approach for reaching the least active people, and those living with (or at risk of) chronic disease. Although many healthcare professional believe they should play a role in supporting people to be more active there are barriers to doing so in terms of time and know-how.

To overcome those barriers, programmes have been developed to bridge health care into community settings with a common goal to optimise the adoption and maintenance of physical activity longterm. These programmes are very diverse according to their location (in- or out- of hospital), their duration, the type and frequency of activity proposed, the level of education of the coaches, or the individual characteristics of each participant.

The studies presented in this symposium explore then the effectiveness of physical activity programmes amongst people with chronic diseases. These explorations are carried out using both quantitative and qualitative approaches, in order to identify as accurately as possible the ins and outs of successful and meaningful support. Holistic consideration of the people’s pathway to an active lifestyle is explored in those studies, embracing particularly the concept of physical literacy.

